# The Emergence of *Bacteroides pyogenes* as a Human Pathogen of Animal Origin: A Narrative Review

**DOI:** 10.3390/microorganisms13061200

**Published:** 2025-05-24

**Authors:** Carola Mauri, Chiara Giubbi, Alessandra Consonni, Elena Briozzo, Elisa Meroni, Francesco Luzzaro, Silvia Tonolo

**Affiliations:** Clinical Microbiology and Virology Unit, “A. Manzoni” Hospital, 23900 Lecco, Italy

**Keywords:** anaerobic infection, animal bite, zoonotic infection, skin and soft tissue infection, MALDI-TOF mass spectrometry identification

## Abstract

*Bacteroides pyogenes* is a Gram-negative obligate anaerobe rod. It is naturally found in the oral microbiome of cats and dogs, which represents a primary source of disease for humans. The present review provides an update on the role of *B. pyogenes* as a pathogen responsible for infections in humans. Indeed, an increasing number of *B. pyogenes* infections have been reported in recent years, including skin and soft tissue infections as well as severe diseases like osteomyelitis, Lemierre’s syndrome, and bloodstream infection. Pre-analytical and analytical phases are crucial to guarantee the isolation of anaerobic bacteria, including *B. pyogenes*. Moreover, the introduction of MALDI-TOF mass spectrometry and 16S rRNA sequencing in clinical microbiology laboratories may be partially responsible for the increasing number of reports of *B. pyogenes* infections. However, the mechanisms underlying the pathogenicity of *B. pyogenes* remain poorly understood and require further investigations. Indeed, despite common antimicrobial susceptibilities, infections frequently persist and require multiple courses of antibiotics. In addition, based on literature data, this review indicates that treatment of skin and soft tissue infections often necessitates surgical procedures and hospitalization.

## 1. Introduction

The genus *Bacteroides* belongs to the Bacteroidaceae family, the order Bacteroidales, the class Bacteroidia, and the phyla Bacteroidota (1 of 41 bacterial phyla), and includes at least fifty species [1]. Bacteria belonging to the genus *Bacteroides* are obligately anaerobic Gram-negative, non-spore-forming, non-motile rods. These bacteria have a critical role as commensal organisms in the human gut but are also involved in human disease, having a potential pathogenic role outside the gut [2]. Among anaerobes, Bacteroides are the most frequently isolated in clinical specimens [3]. In particular, *Bacteroides fragilis* was the first member of the genus recognized in 1898 as a human pathogen [4] and, to date, is still considered the most virulent [3]. *Bacteroides thetaiotaomicron*, *Bacteroides uniformis*, and *Bacteroides vulgatus* are considered other clinically relevant species.

Advances in microbiological diagnostic techniques, including the increasing use of Matrix-Assisted Laser Desorption Ionization–time-of-flight Mass Spectrometry (MALDI-TOF MS), and the use of 16S rRNA sequencing have allowed the increasing isolation of “new” or previously rarely described organisms, including *Bacteroides pyogenes.*

*B. pyogenes* (py.o’ge.nes. Gr. n. pyum pus; Gr. v. gennaio produce; M.L. adj. pyogenes pus-producing) was first described with *Bacteroides suis* from the abscesses and feces of pigs by Benno et al. in 1983 [5] and then isolated from uteri of dairy cows with metritis and from horses’ wounds [6,7,8]. In 1997, Forsblom and colleagues reported the presence of *B. pyogenes* and *Bacteroides tectum* as major components of the anaerobic, Gram-negative, saccharolytic microflora of dogs and their possible role in the development of periodontal disease [9].

Sakamoto et al. studied the *B. pyogenes* genome, concluding that *B. suis* and *B. tectus* are heterotypic synonyms of *B. pyogenes*, showing 100% similarity for the *hsp60* and 16S rRNA genes [10]. Four years later, analysis by whole-genome sequencing of these three strains revealed the diversification of *B. pyogenes* strains isolated from different animals [11]. Sakamoto et al. concluded that further genome analysis will improve our understanding of this species [11]. *B. pyogenes* is included in the *Bacteroides fragilis* group [12].

*B. pyogenes* is usually considered an uncommon but significant human pathogen. However, an increasing number of reports are currently describing its isolation in human clinical specimens, particularly in those collected from wounds [3,13]. Indeed, *B. pyogenes* is a component of the oral microbiota of animals, like dogs and cats [9,14], and can be transmitted to humans through animal bites. The high numbers of domestic dogs and cats in the European Union in 2023, estimated at over 65 million and 75 million, respectively [15,16], highlights the possible growing impact of *B. pyogenes* in the development of human infections.

## 2. Materials and Methods

### 2.1. Search Strategy and Inclusion and Exclusion Criteria

This narrative review aims to summarize all the data on *B. pyogenes* infections in humans published in the literature. Information regarding patients’ demographics, clinical characteristics, site of infection, clinical presentation, and treatment provided were described. There was a particular focus on data regarding microbiological characteristics and methodologies used for identification and antimicrobial susceptibility testing (AST). By using the PubMed/Medline database, searches for relevant articles were performed with the following item: “(Bacteroides pyogenes)”. Studies providing original data, such as case reports and letters to the editor providing information on *B. pyogenes* infections in humans and published up to 31 January 2025 were included in the review. Studies regarding infections and/or colonization in animals were excluded from the analysis. The references of included articles were also examined to identify any studies potentially missed in the initial search.

### 2.2. Data Extraction

After the initial screening, the following data were extracted from each included study: publication year, age and gender of patients, type of infection, contact with animals, treatment (antibiotic therapy and/or surgical procedure), underlying disease, and hospitalization. Other relevant microbiological data were collected: type of sample, method of identification, antimicrobial susceptibility testing profile and co-isolated bacteria.

## 3. Results

### 3.1. Literature Search

The search strategy yielded 42 references; 36 articles were excluded based on exclusion criteria as previously described. Overall, 16 studies were included and analyzed: 9 case reports, 5 letters to the editor and 2 articles, describing a total of 39 cases of infection.

### 3.2. Epidemiology of B. pyogenes Infections

*B. pyogenes* is an obligate anaerobe commensal from the oral cavity of cats and dogs. *B. pyogenes* infections have been described in the United States [17,18] and different countries from Europe [3,13,19,20,21,22], Asia [23,24,25,26,27,28], and Oceania [29,30].

Table 1 reports clinical features of *B. pyogenes* infections described in the literature. Skin and soft tissue infections, mainly associated with animal bites, are the most frequent human infections caused by this microorganism [3,13,17,19,22,23,24,26,29]. *B. pyogenes* can also be responsible for severe diseases, such as bloodstream infections [13,22,23,27], bone infections including osteomyelitis [3,19,20], joint prosthesis infections [21], and intra-abdominal infections [3,25,27]. A case of Lemierre’s syndrome [18], one multiple lung abscess [30], and one urinary tract infection [3] were also described (Table 1 and Table 2). Notably, in some cases, a single patient can develop an infection from *B. pyogenes* in different anatomical sites, as reported by Park and colleagues, who described the presence of *B. pyogenes* in blood and material from a liver abscess [27], or by Chen and colleagues, who identified *B. pyogenes* in blood and purulent exudate from a head injury [23].

The medical history of patients is not always complete and often underlying pathologies are not reported. However, in some cases, underlying diseases were described, with the most frequently reported being diabetes and history of oncology (Table 2 and Supplementary Table S1).

In most cases, *B. pyogenes* infections are described in patients reporting close contact with animals (e.g., bites, licks), confirming that direct contact with animals should be considered the main route of transmission of *B. pyogenes.* Except for the case report by Chen and colleagues describing an infection after a snow leopard bite [23], in all other cases the contact with companion animals (cats and dogs) seems to be the main route of transmission (Table 1 and Table 2). This phenomenon may be linked to the closer contact between companion animals and humans. Nevertheless, in some cases *B. pyogenes* infections seem to be unrelated to animal bites, where contact with animals is not documented in the medical history of the patient or did not occur (Table 1 and Table 2). Minor scratches and bites caused by pets are not always reported to the healthcare facility.

Previous studies on *Pasteurella multocida*, a facultative anaerobic Gram-negative coccobacillus present in the oral and respiratory tracts of cats and dogs, hypothesized that the contamination of a wound with animal fluids or animal air, as well as the ingestion of food contaminated by the animal, may be associated with the onset of opportunistic infections, particularly in patients with comorbidities or in treatment with immunosuppressant [31]. For this reason, future studies on *B. pyogenes* are required to investigate potential transmission mechanisms other than animals’ bites.

Most references describe polymicrobial infections between *B. pyogenes* and other pathogens (Table 2 and Table 3). When more pathogens are detected, the specific contribution of *B. pyogenes* in the development of the infection cannot be conclusively assessed. Anaerobic bacteria, such as *Fusobacterium* spp. and *Pasteurella* spp., are among the coinfecting pathogens most frequently identified. In particular, *P. multocida* is considered responsible for skin and soft tissue infections, but it can be more rarely associated with severe infections such as bone and joint prosthesis infections, endocarditis, sepsis, and meningitis, particularly in immunocompromised hosts [32,33].

### 3.3. Pathogenesis of B. pyogenes Infections

Despite the clinical significance of *B. pyogenes* infections, the mechanisms underlying its pathogenicity remain poorly understood. Usually, limited superficial soft tissue infections and abscesses are reported among healthy individuals. However, immunocompromised patients or those with important comorbidities may develop severe and/or systemic infections.

The pathogenicity of *Bacteroides* spp. includes the presence of a polysaccharide capsule, release of lipopolysaccharide, evasion of host immune response, production of proteolytic enzymes and hemolysin, release of enterotoxin, and aerotolerance [2]. Limited data on specific virulence factors of *B. pyogenes* are currently available; however, its ability to thrive in anaerobic conditions and cause systemic infections suggests the presence of mechanisms helping tissue invasion and immune evasion. *B. pyogenes* produces metabolic products such as succinic acid and acetic acid, which are thought to contribute to its survival in the anaerobic environments of host tissues [34]. It also demonstrates resistance to several host defense mechanisms, including phagocytosis by immune cells. The exact role of *B. pyogenes* in biofilm formation, a critical factor for chronic infection, needs to be better investigated [35]. Furthermore, there is evidence suggesting that *B. pyogenes* may harbor genetic elements conferring resistance to certain antimicrobial agents, complicating treatment regimens.

### 3.4. Diagnosis of B. pyogenes in Humans

Pre-analytical and analytical phases are crucial for guaranteeing the isolation of anaerobic bacteria, including *B. pyogenes*. Anaerobic infections may be underestimated because of difficulties related to their isolation and identification. Similarly to *Pasteurella* spp., *B. pyogenes* should be considered a potential etiological agent of soft tissue infections resulting from scratching, biting, or licking by domestic animals [31].

Proper sample collection is essential and should ideally be performed before the start of antibiotic therapy. Specimens must be free from contamination with commensal microbiota and representative of the infection site (aspirates and tissue biopsies should be preferred to swabs). All samples should be deeply inserted into anaerobic transport media or collected directly in oxygen-free containers. The transport to the laboratory should be at room temperature within two hours from the collection [36].

#### 3.4.1. Identification of *B. pyogenes*

Diagnosis of *B. pyogenes* infection in clinical specimens can be achieved by isolating the pathogen from a culture or using molecular methods.

For culture-based methods, it is essential to provide appropriate conditions for the isolation of anaerobic bacteria, both in terms of culture medium and incubation environment. Specimens should be adequately inoculated on specific culture media, for example, Columbia Agar or Schaedler agar with 5% sheep blood and incubated for 48–72 h in anaerobic conditions (e.g., using anaerobic pouches, boxes, or jars) [3,36].

Table 4 summarizes the methods used for the identification of *B. pyogenes* in previously published reports. The identification of *B. pyogenes* with traditional biochemical methods may be inaccurate due to phenotypically similar species. Indeed, prior to the implementation of MALDI-TOF MS and 16S rRNA sequencing in diagnostics of bacterial infections, the identification of anaerobic microorganisms, such as *B. pyogenes*, was performed using biochemical methods, such as the VITEK-2 ANC card (bioMérieux, Marcy l’Etoile, France) or API Rapid ID 32A (bioMérieux, Marcy l’Etoile, France). Erroneous results of biochemical tests may be responsible for misidentification of the microorganism: *Prevotella oralis* and *Prevotella melaninogenica* were among the species more frequently misidentified [18,21,28,29] (Table 4).

The introduction of MALDI-TOF MS and 16S rRNA sequencing in clinical microbiology laboratories may be partially responsible for the increasing number of reports of *B. pyogenes* infections in last years. The accuracy of MALDI-TOF MS [37] and 16S rRNA gene sequencing [38] to correctly identify anaerobic microorganisms has been widely demonstrated (Table 4). Both techniques have shown good performance in the identification of *B. pyogenes* [3,13,18,21,22,23,24,25,27,28,29], but MALDI-TOF MS is more frequently used in routine diagnostics because of its rapidity and reduced costs. However, to provide a correct identification of all microorganisms [36] and, in particular *B. pyogenes* [27,29], it is fundamental to maintain updated databases of identification instruments. Data libraries for anaerobes should include reference strains as well as clinical isolates corroborated by sequencing methods [36]. Park and colleagues reported a misidentification of *B. pyogenes* with *B. uniformis* using Vitek MS (bioMérieux, Marcy l’Etoile, France) (Table 3 and Table 4) [27].

#### 3.4.2. Antimicrobial Susceptibility of *B. pyogenes*

Culture-based methods are fundamental to guaranteeing the execution of antimicrobial susceptibility testing. Drug susceptibility testing of *B. pyogenes* isolates should be performed according to the most recent International Guidelines of European Committee on Antimicrobial Susceptibility Testing (EUCAST) [39] or the Clinical & Laboratory Standards Institute (CLSI) [40]. The current version of EUCAST Guidelines, version 15.0, provides indications for the interpretation of the results of susceptibility testing of *B. pyogenes* for ampicillin–sulbactam, amoxicillin–clavulanic acid, piperacillin–tazobactam, ertapenem, imipenem, meropenem, clindamycin, and metronidazole, while the use of CLSI criteria offers the possibility to report results of other molecules; for example, moxifloxacin. Moreover, EUCAST Guidelines for the interpretation of amoxicillin–clavulanic acid were first published in Version 13.1 in June 2023. For this reason, most of the studies currently available interpret their results using CLSI criteria.

The use of different international guidelines with different antibiotic breakpoints for the interpretation of antimicrobial susceptibility testing makes the comparison of results between studies difficult. As reported in Table 3 and Table 5, data on antimicrobial susceptibility testing are not always clearly reported. However, in most of the cases, EUCAST criteria were used for the interpretation of the results (*n* = 13) [3], while CLSI criteria were used in five cases [17,22,28,29] and EUCAST with CLSI (for amoxicillin–clavulanate and moxifloxacin) were applied in eight cases [13,19,20]. In three cases, no indication of the criteria used for the interpretation of the results of antimicrobial susceptibility testing were reported [21,24,26].

Antibiotic resistance among *Bacteroides* spp. has increased over the past decade [36,41]. Unlike other *Bacteroides* spp., *B. pyogenes* has been shown to be highly susceptible to most of the molecules usually tested. Cunha and colleagues, in their study on *B. pyogenes* strains from the uteri of cows with metritis, described the presence of a beta-lactam resistance gene (*cfxA2*) in the four isolated tested. Moreover, they observed the presence of the *tetQ* gene, encoding for a ribosomal protection protein conferring resistance to tetracycline, and the *sul2* gene, encoding for a dihydropteroate synthase type-2 and imparting sulfonamide resistance in some of the isolates [8]. Moreover, a study on the genomics of isolates belonging to the *B. fragilis* group excluded the presence of overexpression of the *cfiA* gene in *B. pyogenes*. The *cfiA* gene, responsible for carbapenem resistance, was, however, present in some of the *B. fraglis* isolates tested [42].

As described in Table 5, metronidazole, amoxicillin–clavulanate, and clindamycin were the antibiotics whose activity was most frequently tested against *B. pyogenes* strains. In most of the cases, beta-lactams associated with beta-lactamase inhibitors, carbapenems, clindamycin, and metronidazole demonstrated good in vitro activity against most *B. pyogenes* isolates (Table 5) [3,13,21,22,24]. Moreover, *B. pyogenes* showed a good susceptibility to penicillins [22,26], even though all seven isolates identified by Majewska and colleagues were found to be resistant [3].

## 4. Treatment and Outcomes of *B. pyogenes* Infections

Given the susceptibility of *B. pyogenes* to many classes of antimicrobials, the treatment of infections is often based on beta-lactams, such as amoxicillin–clavulanate, piperacillin–tazobactam and ceftriaxone, and/or metronidazole (Supplementary Table S1). The selection of the appropriate regimen of treatment should take into account the outcome of antimicrobial susceptibility testing, while the duration should be evaluated based on the site of the infection and response to the treatment. Moreover, in most cases, *B. pyogenes* was co-isolated with other pathogens, further hindering the selection of appropriate antibiotic therapy (Table 3).

Despite appropriate antimicrobial therapy, in the presence of animal bites and scratch-related infections, correct wound hygiene and close follow-up are fundamental to avoid complications. In particular, the adequate management of those infections requires the control of their focus. In addition to antimicrobial treatment, surgical procedures, such as incision or debridement, and hospitalization are frequently required to treat *B. pyogenes* infections and guarantee their favorable evolution (Table 2 and Supplementary Table S1).

## 5. Conclusions

The present narrative review summarizes the characteristics of infections by *B. pyogenes* in humans by gathering all the published articles in the PubMed literature.

*B. pyogenes* infections may affect any organ and system but mainly involve skin and soft tissues. In any wound resulting from an animal bite or scratch, primarily cats and dogs, anaerobes like *B. pyogenes* should be suspected as the causative agents of the infection. The contact with the saliva of the animal in the absence of a bite could be sufficient to allow infection with *B. pyogenes*. Careful medical history-taking and microbiological confirmation of the infection are essential for accurate diagnosis and choice of appropriate treatment.

The introduction of MALDI-TOF MS and 16S rRNA sequencing in clinical microbiology laboratories has allowed the identification of species of bacteria previously unassociated with human infections or rarely reported, such as *B. pyogenes*. Although *B. pyogenes* has been shown to be highly susceptible to most of the molecules usually tested in vitro except penicillin, antimicrobial susceptibility testing of the isolated strains is recommended. Despite the susceptibility to antibiotics, the treatment of infections caused by *B. pyogenes* often necessitates surgical procedures, hospitalization, and intravenous therapies. Meticulous monitoring of the infection’s progression is critical to ensure effective clinical management. While the pathogenic mechanisms of other *Bacteroides* spp. have been described, specific investigation on the pathogenic potential of *B. pyogenes* is still required to better understand the mechanisms underlying the development of infections caused by this microorganism.

## Figures and Tables

**Table 1:** Clinical features of *Bacteroides pyogenes* infections reported in the literature, including age, gender, type of infection, contact with animals, and treatment.

Author Year Reference	Age, Gender	Type of Infection	Contact with Animals	Treatment
2025 Chen [23]	33, M	Subcutaneous abscess	Snow leopard bite	Multiple antibiotic therapies and surgical procedure
2024 Lee [30]	55, M	Multiple lung abscesses	Recent exposure to cats and dogs but no known bites	Multiple antibiotic therapies and surgical procedure
2024 Sadhwani [17]	81, F	Intramuscular abscess	Cat bite	Multiple antibiotic therapies and surgical procedure
2023 Vecilla [13] 2023 Vecilla [20] case report regarding #1 2023 Vecilla [19] case report regarding #6	#1 50, M	Jaw osteomyelitis and masseter myositis	Contact with his cat but no bites or scratches	Antibiotic therapy and surgical procedure
	#2 62, F	Cellulitis on the arm	Cat bite	Antibiotic therapy
	#3 79, M	Surgical wound infection (tracheostomy)	Unknown	Antibiotic therapy
	#4 82, M	Bacteremia	Unknown	Antibiotic therapy
	#5 22, M	Ear, nose, and throat infection (fistula)	Unknown	Surgical procedure
	#6 40, M	Osteomyelitis of the second metacarpal with bone destruction of its head	Cat bite	Multiple antibiotic therapies and surgical procedure
	#7 37, F	Cellulitis on the hand	Cat bite	Antibiotic therapy
	#8 57, M	Infected ulcer on the leg	Unknown	Antibiotic therapy
2021 Takahashi [25]	79, F	Appendiceal abscess	No history of animal contact	Multiple antibiotic therapies and surgical procedure
2021 Shenoy [24]	55, M	Diabetic foot infection	Contact with domestic animals such as street dogs, cats, and cattle, without history of any animal bite	Multiple antibiotic therapies and surgical procedures
2021 Majewska [3]	#1 67, M	Soft tissue necrosis within oral cavity and osteomyelitis of the mandible bone	No history of animal contact	Antibiotic therapy and surgical procedure
	#2 70, M	Odontogenic infection, chronic maxillary sinusitis (left) and osteomyelitis of the jaw bone	No history of animal contact	Antibiotic therapy and surgical procedure
	#3 84, F	Phlegmon on the skin and necrotic lesions with fistulas	No history of animal contact	Antibiotic therapy and surgical procedure
	#4 35, M	Trauma: multiple fracture of mandibular bones with displacement	No history of animal contact	Multiple antibiotic therapies and surgical procedure
	#5 67, M	Osteomyelitis	No history of animal contact	Multiple antibiotic therapies and surgical procedures
	#6 73, M	Cholecystitis	No history of animal contact	Antibiotic therapy
	#7 89, F	Phlegmon of the right hand	Cat bite	Antibiotic therapy and surgical procedure
	#8 72, F	Urinary tract infection	No history of animal contact	Antibiotic therapy and surgical procedure
	#9 79, F	Subcutaneous tissue inflammation and phlegmon of the right foot	No history of animal contact	Surgical procedure
	#10 10, F	Skin and soft tissue infection	Dog bite	Not reported
	#11 35, F	Skin and soft tissue infection	Dog bite	Not reported
	#12 39, F	Skin and soft tissue infection	Animal contact not clear (incomplete medical history)	Not reported
	#13 55, M	Skin and soft tissue infection	Animal contact not clear (incomplete medical history)	Not reported
2021 Goggin [18]	7, M	Lemierre’s syndrome	Contact with 3 domestic dogs, that often slept with him and roused him in the morning by licking his head, neck, and ears. No history of any animal bite	Multiple antibiotic therapies and surgical procedure
2019 Gual-de-Torrella [21]	53, F	Abscess from the surgical wound of primary knee arthroplasty of the patella. Eschar of the surgical wound	Contact with her domestic dog, without history of bites or scratches (patient has an open surgical wound)	Multiple antibiotic therapies and surgical procedure
2018 Umemura [26]	53, F	Abscess on the left foot	Cat bite	Multiple antibiotic therapies and surgical procedure
2016 Park [27]	77, F	Bacteremia secondary to liver abscess	No history of animal bite	Antibiotic therapy and surgical procedure
2016 Lau [29]	#1 46, F	Skin and soft tissue infection	Dog bite on the left index finger	Multiple antibiotic therapies and surgical procedure
	#2 46, M	Skin and soft tissue infection	Cat bite on the right lower leg	Multiple antibiotic therapies and surgical procedures
	#3 69, F	Abscess on the right forearm	Cat bite	Multiple antibiotic therapies and surgical procedure
	#4 47, F	Skin and soft tissue infection	Dog bite on the left index finger	Multiple antibiotic therapies and surgical procedure
	#5 76, M	Skin and soft tissue infection	Contact with his two dogs without history of any animal bite	Multiple antibiotic therapies and surgical procedures
	#6 10, M	Skin and soft tissue infection	Dog bite on the right-hand 1st webspace	Multiple antibiotic therapies and surgical procedures
	#7 67, M	Joint infection	Cat bite on the left-hand 2nd metacarpophalangeal joint	Multiple antibiotic therapies and surgical procedures
2016 Kim [28]	51, F	Bloodstream infection	No history of animal contact	Multiple antibiotic therapies
2011 Madsen [22]	60, M	Skin and soft tissue infection, and bacteremia	Cat bite on the left wrist	Multiple antibiotic therapies and surgical procedures

Abbreviations: F: Female; M: Male.

**Table 2:** Summary of *Bacteroides pyogenes* infections reported in the literature.

Gender	21 M; 18 F
Age	Range: 7–89 Average: 55.6
Type of infection	Skin and soft tissue infection, *n* = 24 Bone infection, *n* = 5 Intra-abdominal infection, *n* = 4 Bloodstream infection, *n* = 1 Intramuscular abscess, *n* = 1 Joint prosthesis infection, *n* = 1 Lemierre’s syndrome, *n* = 1 Lung abscess, *n* = 1 Urinary tract infection, *n* = 1
Contact with animal	Yes, *n* = 22 (16 animal bites: cats, *n* = 10; dogs, *n* = 5; snow leopard, *n* = 1; 6, no history of animal bite); No history of animal contact, *n* = 10 Animal contact not clear or incomplete medical history, *n* = 7
Monomicrobial infection	9 samples
Polymicrobial infection	30 samples (6 patients with more than one samples; at least 1 was polymicrobial) Other anaerobic bacteria, *n* = 24 (*Pasteurella* spp. *n* = 10) Only aerobic bacteria, *n* = 6
Underlying disease	Medical history not always complete, *n* = 10 No underlying disease, *n* = 11 Diabetes mellitus, *n* = 6 Oncologic history, *n* = 3 Diabetes mellitus and oncologic history, *n* = 3 Hepatitis infection, *n* = 2 Recent surgery, *n* = 1 Cardiac disease, *n* = 1 Systemic Lupus Erythematosus, *n* = 1
Hospitalization	Yes, *n* = 31 (3 patients in ICU wards) No, *n* = 8 (Range: 2–61 days, average: 16 days)
Treatment	Antibiotic and surgical reatment, *n*= 26 Only antibiotic treatment, *n* = 7 Only surgical treatment *, *n* = 2 Treatment not reported, *n* = 4

Abbreviations: F: Female; M: Male. * Information regarding antibiotic therapy after surgical treatment was not reported.

**Table 3:** Laboratory features of *Bacteroides pyogenes* infections reported in the literature, including type of infection, identification method used, antimicrobial susceptibility, and co-pathogens isolated.

Author Year Reference	Type of Sample	Method of Identification	Antibiotic Resistance Phenotype (MIC mg/L)	Co-Isolated Bacteria
2025 Chen [23]	Purulent exudate	16S rRNA sequencing	NR	*Fusobacterium necrophorum*; *Pasteurella multocida*
	Venous blood			
2024 Lee [30]	Lung abscesses	NR	AST not performed because it was expected to be susceptible to metronidazole	Different bacteria for 4 years. In the sample with *B. pyogenes*: *Fusobacterium nucleatum* and mixed anaerobes
2024 Sadhwani [17]	Intraoperative sample from abscess	NR	AMC-susceptible	*Pasteurella multocida*
2023 Vecilla [13] 2023 Vecilla [20] case report regarding #1 2023 Vecilla [19] case report regarding #6	#1 Pus from masseter muscle abscess	MALDI-TOF mass spectrometry	AMC-, CLI-, IMP-, MEM-, MTZ-, MXF-, TZP-susceptible	No (monomicrobial)
	#2 Swab from wound	MALDI-TOF mass spectrometry	AMC-, CLI-, IMP-, MEM-, MTZ-, MXF-, TZP-susceptible	No (monomicrobial)
	#3 Swab from surgical wound	MALDI-TOF mass spectrometry	AMC-, CLI-, IMP-, MEM-, MTZ-, MXF-, TZP-susceptible	*Proteus mirabilis*, *Gemella* *morbillorum*, *Streptococcus mitis*, and *Corynebacterium amycolatum*
	#4 Blood culture	MALDI-TOF mass spectrometry	AMC-, CLI-, IMP-, MEM-, MTZ-, MXF-, TZP-susceptible	No (monomicrobial)
	#5 Pus from fistula	MALDI-TOF mass spectrometry	AMC-, CLI-, IMP-, MEM-, MTZ-, MXF-, TZP-susceptible	*P. mirabilis*, *Klebsiella oxytoca*, and *Streptococcus constellatus*
	#6 Intraoperatively taken tissue	MALDI-TOF mass spectrometry	AMC-, CLI-, IMP-, MEM-, MTZ-, MXF-, TZP-susceptible	*Pasteurella multocida*
	#7 Pus from abscess	MALDI-TOF mass spectrometry	AMC-, CLI-, IMP-, MEM-, MTZ-, MXF-, TZP-susceptible	*Pasteurella multocida*
	#8 Swab from a wound	MALDI-TOF mass spectrometry	AMC-, CLI-, IMP-, MEM-, MTZ-, MXF-, TZP-susceptible	*Pseudomonas aeruginosa* and *Prevotella intermedia*
2021 Takahashi [25]	Intraoperative appendiceal abscess obtained by intra-abdominal drainage	MALDI-TOF mass spectrometry Biochemical test (identified as *Prevotella melaninogenica*) 16S rRNA sequencing	NR	*Escherichia coli*; *Bacteroides thetaiotaomicron*; *Peptostreptococcus micros*
2021 Shenoy [24]	Intraoperative soft tissue specimens	MALDI-TOF mass spectrometry Biochemical test (identified as *Prevotella oralis*) 16S rRNA sequencing	CLI-, IPM-, MEM-, MTZ-, PIP-susceptible. Β-lactamase-negative	MRSA; *Streptococcus dysgalactiae*
2021 Majewska [3]	#1 Pus from an abscess	MALDI-TOF mass spectrometry	AMC-, IPM-, MTZ-susceptible; CLI-, PEN-resistant	*Eikenella corrodens*, *Enterococcus faecalis*; *Streptococcus pneumoniae*
	#2 Swab from the alveolar jaw	MALDI-TOF mass spectrometry	AMC-, CLI-, IPM-, MTZ-susceptible; PEN-resistant	*Enterococcus faecalis*; *Streptococcus parasanguinis*
	#3 Wound swab	MALDI-TOF mass spectrometry	AMC-, CLI-, IPM-, MTZ-susceptible; PEN-resistant	*Staphylococcus epidermidis*
	#4 Intraoperative tissue	MALDI-TOF mass spectrometry	AMC-, CLI-, IPM-, MTZ-, PEN-susceptible	*Fusobacterium nucleatum*; *Streptococcus anginosus*
	#4 Transtracheal aspirate	MALDI-TOF mass spectrometry		No (monomicrobial)
	#5 Intraoperatively taken tissue	MALDI-TOF mass spectrometry	AMC-, CLI-, IPM-, MTZ-, PEN-susceptible	*Finegoldia magna*; *Proteus mirabilis*
	#6 Bile	MALDI-TOF mass spectrometry	AMC-, CLI-, IPM-, MTZ-, PEN-susceptible	*Clostridium perfringens*; *Escherichia coli*
	#7 Pus from an abscess	MALDI-TOF mass spectrometry	AMC-, CLI-, IPM-, MTZ-susceptible; PEN-resistant	No (monomicrobial)
	#8 Urine (ureteral catheter)	MALDI-TOF mass spectrometry	AMC-, CLI-, IPM-, MTZ-, PEN-susceptible	*Veilonella atypica*
	#9 Wound swab	MALDI-TOF mass spectrometry	AMC-, CLI-, IPM-, MTZ-, PEN-susceptible	No (monomicrobial)
	#9 Wound swab (29 days later)	MALDI-TOF mass spectrometry	AMC-, CLI-, IPM-, MTZ-susceptible; PEN-resistant	MRSA; *Enterococcus faecalis*; *Pseudomonas aeruginosa*; *Alcaligenes faecalis*
	#10 Wound swab	MALDI-TOF mass spectrometry	AMC-, CLI-, IPM-, MTZ-, PEN-susceptible	*Fusobacterium nucleatum*; *Pasteurella multocida*
	#11 Wound swab	MALDI-TOF mass spectrometry	AMC-, CLI-, IPM-, MTZ-susceptible; PEN-resistant	*Peptostreptococcus harei*; *Cutibacterium acnes*
	#12 Wound swab	MALDI-TOF mass spectrometry	AMC-, CLI-, IPM-, MTZ-susceptible; PEN-resistant	*Fusobacterium nucleatum*; *Staphylococcus aureus*
	#13 Wound swab	MALDI-TOF mass spectrometry	AMC-, CLI-, IPM-, MTZ-, PEN-susceptible	*Pasteurella canis*
2021 Goggin [18]	Intraoperative samplefrom abscess	Biochemical test (identified as *Prevotella oralis*) 16S rRNA sequencing	NR	*Alcaligenes xylosoxidans* spp. *xylosoxidans*, *Gemella* species, *Granulicatella elegans*, *Staphylococcus epidermidis*, non-hemolytic diphtheroides
2019 Gual-de-Torrella [21]	Four intraoperative specimens (osteoarticular biopsy, wound exudate, joint fluid, synovial tissue) and prosthesis	MALDI-TOF mass spectrometry	AMC-, CLI-, IPM-, MTZ-, PEN-, TZP-susceptible	*Peptostreptococcus canis*
	Prosthesis, osteoarticular biopsy, joint fluid (after 10 days of treatment)	MALDI-TOF mass spectrometry (recovered only from the prosthesis culture)	NR	No (monomicrobial)
2018 Umemura [26]	Pus discharge from abscess	MALDI-TOF mass spectrometry	PEN-, cephem-, and new quinolone-susceptible	No, but minocycline (3 weeks of therapy) might have killed other pathogens before the culture
2016 Park [27]	Blood culture	MALDI-TOF mass spectrometry (identified as *Bacteroides pyogenes* by Bruker system; identified as *Bacteroides uniformis* by VITEK MS) Biochemical test for blood culture sample (identified as *Prevotella oralis*) 16S rRNA sequencing for blood culture sample	NR	No (monomicrobial)
	Pus from liver abscess			*Klebsiella pneumoniae*
2016 Lau [29]	#1 Wound swab	MALDI-TOF mass spectrometry Biochemical test (performed only on 5 isolates, identified as *Prevotella oralis*, *n* = 4; and *Prevotella melaninogenica*, *n* = 1) 16S rRNA sequencing	NR	No (monomicrobial)
	#2 Wound swab		NR	*Pasteurella multocida*
	#3 Wound swab		NR	No (monomicrobial)
	#4 Wound swab		NR	*Pasteurella canis* *Pasteurella stomatis* *Staphylococcus pseudointermedius*
	#5 NA		AMC 0.094, MTZ 0.50, MXF 0.047, PEN 0.016, TZP 0.064	*Staphylococcus aureus* *Morganella morganii* *Atopobium deltae*
	#6 Wound swab		NR	*Pasteurella dagmatis*
	#7 Wound swab		AMC 0.032, MTZ 0.025, MXF 0.047, PEN 0.016, TZP 0.016	No (monomicrobial)
2016 Kim [28]	Blood culture	Biochemical test (identified as *Prevotella oralis*) 16S rRNA sequencing	CLI > 256, CRO 0.064, MEM 0.016, MTZ 0.5, PEN 0.032, TZP 0.016	No (monomicrobial)
2011 Madsen [22]	Pus discharge from abscess	Biochemical test (identified as *Bacteroides capillosus*) 16S rRNA sequencing	CLI, MEM, MTZ, PEN, TZP susceptible	*Pasteurella multocida*
	Blood culture			No (monomicrobial)

Abbreviations: NR: not reported; NA: not available; AMC: amoxicillin–clavulanate; CLI: clindamycin; CRO: ceftriaxone; IPM: imipenem; MEM: meropenem; MRSA: methicillin-resistant *Staphylococcus aureus*; MTZ: metronidazole; MXF: moxifloxacin; PEN: penicillin; PIP: piperacillin; TZP: piperacillin–tazobactam. Cases with absence of bacterial growth in the aforementioned studies were omitted.

**Table 4:** Methods used for *Bacteroides pyogenes* identification previously reported in the literature.

Type of Identification	N. of Test	Results	Instrument
MALDI-TOF mass spectrometry	33	*Bacteroides pyogenes* was misidentified as: *Bacteroides uniformis* (*n* = 1, by Vitek MS) In 5 samples, the Vitek MS instrument was not able to identify the pathogen	Bruker (*n* = 17) Vitek MS (*n* = 21) Not Reported (*n* = 1)
Biochemical test	11	*Bacteroides pyogenes* was misidentified as: *Prevotella oralis* (*n* = 8) *Prevotella melaninogenica* (*n* = 2) *Bacteroides capillosus* (*n* = 1)	VITEK 2 ANC Card (*n* = 9) API Rapid ID 32A anaerobe identification system (*n* = 2)
16S rRNA sequencing	14	*Bacteroides pyogenes* Matching score: 94–100%	Sanger Sequencing (*n* = 10) Next-Generation Sequencing (*n* = 2) Not Reported (*n* = 2)

**Table 5:** Antimicrobial susceptibility testing results of *Bacteroides pyogenes* infections reported in the literature.

Antibiotic	N. of Test Performed	Susceptible N. (%)
Amoxicillin–clavulanate	25	25 (100%)
Clindamycin	25	23 (92%)
Imipenem	23	23 (100%)
Meropenem	11	11 (100%)
Metronidazole	28	28 (100%)
Moxifloxacin	10	10 (100%)
Penicillin	19	12 (63.2%)
Piperacillin–tazobactam	13	13 (100%)

## Data Availability

No new data were created or analyzed in this study. Data sharing is not applicable to this article.

## Supplementary Materials

The following supporting information can be downloaded at: https://www.mdpi.com/article/10.3390/microorganisms13061200/s1, Supplementary Table S1: Clinical features of *Bacteroides pyogenes* infections reported in the literature, including age, gender, clinical presentation, underlying illness and risk factors, medical history, contact with animals, treatment, and outcome.
