# Supplementary material for: The Emergence of Bacteroides pyogenes as a Human Pathogen of Animal Origin: A Narrative Review
Source: Microorganisms. 2025 May 24;13(6):1200. doi: 10.3390/microorganisms13061200 (PMC12194902; doi:10.3390/microorganisms13061200)
Supplement: Supplementary file 1 [file microorganisms-13-01200-s001.zip › microorganisms-3544694-supplementary.pdf]

Table S1. Clinical features of *Bacteroides pyogenes* infections reported in the literature, including age, gender, clinical presentation, underlying illness and risk factors, medical history, contact with animals, treatment, and outcome.

Author Year Reference	Age, gender	Clinical presentation	Underlying illness and risk factors	Medical history	Contact with animals	Treatment	Outcome
2025 Chen [23]	33, M	Head injuries, headache, fever, and palpitations	NR	NR	Snow leopard bite	At admission: AMC. At wound debridement and suturing, MEM was started.	Patient discharged after 20 days
2024 Lee [30]	55, M	Multiple lung abscesses with 3-month history of productive cough, night sweats, and 5 kg weight loss	Chronic hepatitis B complicated by cirrhosis; splenectomy for autoimmune thrombocytopenia	Poor dentition and periodontal disease	Recent exposure to cats and dogs but no known bites	Various therapies during 4 years of treatment. Latest therapies (probably healing): 7 months of MTZ (400 mg 3 times a day) and 13 months of moxifloxacin (400 mg OD)	Resolution of the abscesses after 4 years of multiple investigations and antimicrobial treatments
2024 Sadhwani [17]	81, F	Left calf pain, swelling, erythema, and fevers	No underlying disease	Deep vein thrombosis, pulmonary embolism, and ischemic stroke without residual deficit	Cat bite	At home: AMC and SXT At admission: intravenous (IV) vancomycin and cefepime (four days) and surgical debridement. Following the discharge: oral AMC (875 mg 2 times a day for 4 weeks).	Resolution of the pain and healing of the incision

 https://doi.org/10.3390/microorganisms13061200

2023 Vecilla [13]<br><br>2023 Vecilla [20] case report regarding #1<br><br>2023 Vecilla [19] case report regarding #6	#1<br>50, M	Right hemiface inflammation and trismus with no other associated symptoms (jaw osteomyelitis and masseter myositis)	No underlying disease	Dental extraction of the last molar of the right lower jaw three months earlier, in the context of dental phlegmon. Corticosteroid treatment.	Contact with his cat but no bites or scratches	Surgical debridement and AMC + MTZ for 8 weeks	Patient discharged after 5 days
	#2<br>62, F	Cellulitis on the arm	Biliary cirrhosis	NR	Cat bite	AMC	NR
	#3<br>79, M	Surgical wound infection (tracheostomy)	DM, alcohol consumption, and malignancy	NR	Unknown	AMC	NR
	#4<br>82, M	Bacteremia	DM	NR	Unknown	TZP	NR
	#5<br>22, M	Ear, nose, and throat infection (fistula)	Recent surgery	NR	Unknown	No follow-up after drainage	NR
	#6<br>40, M	Osteomyelitis of the second metacarpal with bone destruction of its head	Moderate alcohol consumption	No history of interest	Cat bite	At home: multiple antibiotic therapy.<br>During the hospitalization: resection of the second metacarpal head) and AMC (IV, 2 g/200 mg/8 h, for 18 days) and	Clinical follow-up of the patient showed reports of improvement and disappearance of pain.

						MTZ (OS, 500 mg/8 h for 14 days). Discharge medication consisted of LVX (750 mg/24 h) and MTZ (250 mg/8 h) for 2 weeks and then MXF (400 mg) for 2 more weeks.	
	#7 37, F	Cellulitis on the hand	No underlying disease	NR	Cat bite	AMC	NR
	#8 57, M	Infected ulcer on the leg	DM and malignancy	NR	Unknown	AMC	NR
2021 Takahashi [25]	79, F	Lateral and lower right abdominal pain; CT indicated a rupture and abscess of the appendix	High blood pressure; arrhythmia	Cholecystectomy and lobectomy for right lower lung cancer	No history of animal contact	On admission: TZP (4.5 g/6 h / IV). Changed to AMX (250 mg, 3 times a day) and then to AMC (250 mg, 3 times a day)	Patient discharged after 12 days
2021 Shenoy [24]	55, M	Fever and 2cm x 2cm left foot ulcer associated with bluish discoloration and wet gangrene of the fourth toe	DM for 20 years	Two years previously, complained of diabetic foot and wet gangrene of the fifth toe	Contact with domestic animals such as street dogs, cats and cattle, without history of any animal bite	On admission: FEP (500 mg BD) and LVX (500 mg OD) and surgical debridement. After 2 days, second surgical debridement and amputation of the fourth left toe	After 10 days of hospitalization, was discharged with oral CLI 300 mg BD for 5 days. Healthy at the follow-up visit after 1 month

2021 Majewska [3]	#1 67, M	Extensive, chronic soft tissue necrosis within oral cavity, osteomyelitis of the mandible bone	Oncologic history, immunosuppression, prosthesis	Three years earlier: carcinoma of the lower lip with reconstruction plate and radiation therapy	No history of animal contact	AMP and GEN	Patient discharged after 28 days
	#2 70, M	Odontogenic infection, chronic maxillary sinusitis (left) and osteomyelitis of the jaw bone	No underlying disease	NR	No history of animal contact	CLI	Patient discharged after 3 days
	#3 84, F	Phlegmon on the skin (neck and anterior chest wall), necrotic lesions with fistulas, primary infection in the oral cavity	No underlying disease	NR	No history of animal contact	MTZ and VAN	Patient discharged after 36 days
	#4 35, M	Trauma: multiple fracture of mandibular bones with displacement	No underlying disease	NR	No history of animal contact	AMP and CLI (for 7 days), then CRO and MZT	Patient discharged after 23 days

	#5<br>67, M	Forefoot necrosis, edema, bubbles filled with gas, osteomyelitis	DM	NR	No history of animal contact	MTZ and CLI.<br>Amputation of the second and third fingers of the right foot followed by wound surgical debridement (3 times).	Repeated hospital admissions in short intervals
	#6<br>73, M	Cholecystolithiasis	No underlying disease	NR	No history of animal contact	CRO	Patient discharged after 10 days
	#7<br>89, F	Phlegmon of the right hand after cat bite	No underlying disease	NR	Cat bite	AMC and AZI	Patient discharged after 3 days
	#8<br>72, F	Bladder carcinoma	Oncologic history, DM	NR	No history of animal contact	CFX (intraoperative)	Patient discharged after 22 days
	#9<br>79, F	Subcutaneous tissue inflammation and phlegmon of the right foot	No underlying disease	NR	No history of animal contact	NR	Patient discharged after 61 days
	#10<br>10, F	NR	NR	NR	Dog bite	NR	NR
	#11<br>35, F	NR	NR	NR	Dog bite	NR	NR
	#12<br>39, F	NR	NR	NR	Animal contact not clear (incomplete	NR	NR

					medical history)		
	#13 55, M	NR	NR	NR	Animal contact not clear (incomplete medical history)	NR	NR
2021 Goggin [18]	7, M	Persistent fever over two weeks. Tender 4x4 cm mass posterior to the right ear, tympanic membrane perforation with purulent otorrhea. CT showed right-side bone erosion, neck abscess, bilateral pulmonary septic emboli (Lemierre's syndrome).	Healthy and fully immunized	None aside from a history of episodic right-sided ear infections associated with drainage since the age of 4	Contact with 3 domestic dogs, that often slept with him and roused him in the morning by licking his head, neck, and ears. No history of any animal bite.	On admission: VAN and CRO. Surgical removal of a large cholesteatoma and abscess drainage. Therapy changed to VAN, CAZ and MTZ (IV for 3 weeks) followed by oral AMC (3 weeks).	Favorable evolution after treatment but conductive hearing loss due to ossicular damage from cholesteatoma and removal during surgery
2019 Gual-de-Torrella [21]	53, F	Fever and abscess from the surgical wound of primary knee arthroplasty	Systemic Lupus Erythematosus	Patient with an arthroplasty of the patella	Contact with her domestic dog, without history of bites	On admission: VAN and CAZ IV. Changed to PEN IV and RIF.	Favorable evolution after 6 weeks of antibiotic

		of the patella. Eschar of the surgical wound.			or scratches, but the patient has an open surgical wound	The prosthesis was removed and an antibiotic-coated spacer with GEN and CLI was implanted.	treatment. Implantation of a new prosthesis after 6 months.
2018 Umemura [26]	53, F	Afebrile, abscess on the left foot caused by cat bite	NR	NR	Cat bite on the left foot	At home: MIN (200 mg/d for 3 weeks). Changed to sitafloxacin (100 mg/d oral) due to history of drug eruption in response to PEN	The abscess and wound completely healed after 3 weeks of quinolone therapy and two more incisions (with negative cultures)
2016 Park [27]	77, F	Epigastric discomfort, fever, and chills. CT showed a liver abscess, cholecystitis, and radiolucent stones of the common bile duct.	NR	Hypertension and atrial fibrillation combined with hypertrophic cardiomyopathy. Warfarin therapy.	No history of animal bite	After blood culture collection: TZP (2.25 g every 6h)	Patient discharged after 25 days
2016 Lau [29]	#1 46, F	NR		NR	Dog bite on the left index finger	At home: IM CRO. During the hospitalization: IV TZP and oral AMC. Surgical debridement, two	Patient discharged after 5 days

						washouts, and a full-thickness skin graft were necessary.	
	#2<br>46, M	NR		NR	Cat bite on the right lower leg	At home: oral DCX and IM CRO. During the hospitalization: IV TZP and oral AMC. Multiple surgical debridement and washouts were necessary. Also, a split skin graft was performed.	Patient discharged after 14 days
	#3<br>69, F	Abscess on the right forearm	No clinical information detailed, only 3 patients had DM	NR	Cat bite on the right forearm	At home: oral cephalexin, flucoxacillin, AMC, and IM CRO. During the hospitalization: IV TZP and oral AMC. Incision, drainage, and surgical debridement were performed.	Patient discharged after 2 days
	#4<br>47, F	NR		NR	Dog bite on the left index finger	The patient was treated with IV TZP and oral AMC. She also underwent surgical debridement and washout.	Patient discharged after 3 days
	#5<br>76, M	Trauma to toe		NR	Contact with his two dogs without a history of any animal bite	The patient underwent multiple antibiotic therapy: IV TZP, IV flucloxacillin, and IV CIP, then oral AMC and CIP. The left forefoot of the patient was amputated. Then the patient underwent revision of stump and washout.	Patient discharged after 19 days

	#6<br>10, M	NR		NR	Dog bite on the right-hand 1st webspace	The patient was treated with IV TZP and oral AMC. For surgery, he underwent two cycles of incision, drainage, and washout.	Patient discharged after 5 days
	#7<br>67, M	NR		NR	Cat bite on the left-hand 2nd metacarpophal angeal joint	At home: oral DCX, AMX. The patient was treated with IV TZP for 6 weeks. He received multiple surgical debridement and washout; finally, the flap was closed.	Patient discharged after 9 days
2016<br>Kim<br>[28]	51, F	Dyspnea and abdominal pain. Altered mental state. Generalized edema, abdominal distension, jaundice, and body weight of 45 kg.	NR	Liver cirrhosis due to a hepatitis C viral infection and chronic alcohol abuse	No history of animal contact	At admission: CRO (IV 1g 2 times a day). Therapy implemented with TZP (IV 4,5 g 4 times a day).	Patient's fever returned to normal two days after infusion of TZP
2011<br>Madsen<br>[22]	60, M	Thoracic pain and general malaise. Hand red and swollen around the wound.	NR	Prior heroin addiction, lung cancer treated surgically 2 years earlier with left pneumonectomy and adjuvant chemotherapy	Cat bite on the left wrist	After the consultation at the emergency room: DCX (1g four times a day, OS). After the hospitalization: PEN (1 MU four times a day). Several revisions of the hand and wrist were necessary. Vacuum-assisted closure treatment was also performed.	Patient discharged after 9 days

Abbreviations: DM, diabetes mellitus; MOF, multiple organ failure; NA: not available; CT: computer tomography; IM: intramuscular; IV: intravenous; AMC: amoxicillin–clavulanate; AMP: ampicillin; AMX: amoxicillin; AZI: azythromycin; CAZ: ceftazidime; CFX: cefuroxime; CIP: ciprofloxacin; CLI: clindamycin; CRO: ceftriaxone; DCX: dicloxacillin; FEP: cefepime; GEN: gentamycin; LVX: levofloxacin; MIN: minocycline; MTZ: metronidazole; PEN: penicillin; RIF: rifampicin; SXT: trimethoprim/sulfamethoxazole; TZP: piperacillin–tazobactam; VAN: vancomyci
